# Supercolonial structure of invasive populations of the tawny crazy ant *Nylanderia fulva* in the US

**DOI:** 10.1186/s12862-018-1336-5

**Published:** 2018-12-29

**Authors:** Pierre-André Eyer, Bryant McDowell, Laura N. L. Johnson, Luis A. Calcaterra, Maria Belen Fernandez, DeWayne Shoemaker, Robert T. Puckett, Edward L. Vargo

**Affiliations:** 1 Department of Entomology, 2143 TAMU, Texas A&M University, College Station, TX 77843-2143 USA; 2 Fundación para el Estudio de Especies Invasivas (FuEDEI) and CONICET, Bolívar 1559, B1686EFA Hurlingham, Buenos Aires Argentina; 3 Department of Entomology and Plant Pathology, University of Tennessee, Knoxville, TN 37996-4560 USA

**Keywords:** Invasive species, Mating system, Colony structure, Supercolonies, Social insects, Ants

## Abstract

**Background:**

Social insects are among the most serious invasive pests in the world, particularly successful at monopolizing environmental resources to outcompete native species and achieve ecological dominance. The invasive success of some social insects is enhanced by their unicolonial structure, under which the presence of numerous queens and the lack of aggression against non-nestmates allow high worker densities, colony growth, and survival while eliminating intra-specific competition. In this study, we investigated the population genetics, colony structure and levels of aggression in the tawny crazy ant, *Nylanderia fulva*, which was recently introduced into the United States from South America.

**Results:**

We found that this species experienced a genetic bottleneck during its invasion lowering its genetic diversity by 60%. Our results show that the introduction of *N. fulva* is associated with a shift in colony structure. This species exhibits a multicolonial organization in its native range, with colonies clearly separated from one another, whereas it displays a unicolonial system with no clear boundaries among nests in its invasive range. We uncovered an absence of genetic differentiation among populations across the entire invasive range, and a lack of aggressive behaviors towards conspecifics from different nests, even ones separated by several hundreds of kilometers.

**Conclusions:**

Overall, these results suggest that across its entire invasive range in the U.S.A., this species forms a single supercolony spreading more than 2000 km. In each invasive nest, we found several, up to hundreds, of reproductive queens, each being mated with a single male. The many reproductive queens per nests, together with the free movement of individuals between nests, leads to a relatedness coefficient among nestmate workers close to zero in introduced populations, calling into question the stability of this unicolonial system in which indirect fitness benefits to workers is apparently absent.

**Electronic supplementary material:**

The online version of this article (10.1186/s12862-018-1336-5) contains supplementary material, which is available to authorized users.

## Background

Understanding the evolutionary factors affecting population structure and ecological assemblage is a central question in ecology. This question is especially complex in the context of biological invasions, as introductions usually prompt severe shifts in genetic structure and life history traits of invasive species, and profoundly disturb the ecological community of native species [1, 2]. Population bottlenecks associated with founder events following introductions reduce genetic diversity and may lead to inbreeding, while novel abiotic and biotic pressures in invaded environments require a rapid and efficient response to the new selective forces [3]. Uncovering the mechanisms by which biological invasions induce post-introduction phenotypic changes in life history traits that allow invasive species to overcome the loss of genetic diversity and the reduced adaptive potential to successfully establish and achieve local dominance in a new environment remain important areas of study [4–6].

Social insects are among the most abundant and successful species at invading terrestrial environments. Despite the taxonomic diversity of invasive species, most of them share life history traits that may facilitate their introduction and dominance in new ecosystems [2]. Among these, the invasion success of social insects is often associated with a unicolonial social system, under which the absence of aggressive behavior towards non-nestmates allows free mixing of individuals (workers, brood and queens) among geographically distant nests [7]. Unicoloniality reduces intra-specific competition; this may allow high worker densities and an increased colony growth and survival due to the presence of many reproductive queens within nests. This social organization allows invasive populations to efficiently monopolize environmental resources and rapidly outcompete native species to achieve local dominance [2, 8].

Unicoloniality is a common trait in invasive ant species and several hypotheses have been proposed to account for its evolution in introduced populations [9, 10]. A first hypothesis posits that the loss of genetic diversity in bottlenecked populations lowers nestmate recognition reducing differentiation between colonies. If nestmate recognition is based on heritable cues [11–13], the overall loss of genetic diversity in introduced populations reduces the diversity at the recognition locus (or loci) and homogenizes recognition templates among colonies. Ultimately, if polymorphism at the recognition locus (or loci) is lost, unicoloniality could arise through the inability of workers to discriminate against non-nestmate conspecifics [2, 14, 15]. A second hypothesis suggests that high nest density in the introduced range has selected for reduced nestmate recognition to avoid recurrent fights with their neighbors [9, 16]. The relaxed environmental pressures in the introduced range often lead to high nest densities and increase the rates of encountering non-nestmate conspecifics [17]. This could have selected for lower recognition cues if nonaggressive neighboring colonies attain higher worker number and outcompete aggressive ones [18–20]. A third hypothesis proposes that the species is already polydomous and polygynous, forming small supercolonies in its native range [9, 21]. It suggests that the absence of conspecific competition in the introduced range has enabled the invasive colony to grow extremely large [21]. This scenario requires minimal evolutionary changes, since only the extent of the supercolony changes but not the behavior of workers. Whatever the evolutionary forces triggering unicoloniality [2, 9, 10], the loss of aggressive behavior toward non-nestmates results in the development of supercolonies, a social organization formed from a network of several interconnected nests without clear boundaries between them [22].

Supercolonies may extend across large geographic distances, with populations ultimately consisting of a single, vast supercolony with no aggression towards colony-mates [2, 23–27]. In these populations, the combination of free exchange of individuals between nests and the occurrence of hundreds or even thousands of reproductive queens per nest results in extremely low relatedness between colony-mates that often approaches zero [28–33]. However, studies on a broader geographical scale reveal genetic differentiation may exist such that the entire population comprises several supercolonies [9, 21, 31, 34–37]. The presence of several supercolonies or limited dispersal abilities within a supercolony may reduce gene flow between nests [33], and restore relatedness among colony-mates or lead to hot spots of locally elevated relatedness between nestmates within supercolonies [38].

The tawny crazy ant, *Nylanderia fulva*, is native to South America from Brazil to Argentina along the border of Uruguay and Paraguay [39]. This species has been introduced into Peru, Colombia and the Caribbean [39, 40], and recently was documented in the U.S.A., rapidly spreading across Florida, southern Mississippi, southern Louisiana and Texas [41, 42]. From the 1950’s to the 2000’s, this species was only reported in Florida (under the synonym *Paratrechina pubens).* It was later uncovered in Texas during a sudden outbreak in 2002; and more recently in Mississippi in 2009 [42]. Field observations in the U.S.A. introduced range revealed that populations consist of dense networks of polygynous nests (0 to 5 reproductive queens, [43]), with ants freely moving among them without any aggression between non-nestmates [44, 45]. No nuptial flights have been observed in the invasive range, suggesting that the invasion front advances by nest fission (or budding), where queens establish new nests with the help of workers within walking distance of the natal nest [41, 46]. However, these studies only used field observations with limited behavioral tests; genetic studies are lacking to clearly determine the population genetics, colony structure and aggression patterns of the tawny crazy ant within its introduced range, and no information is available from the species’ native range.

In this study, we conducted large-scale genetic and behavioral analyses of the invasive tawny crazy ant. We first investigated patterns of population genetic structure within its native and introduced ranges to estimate the extent of the genetic diversity loss stemming from the founder effect following its introduction in the U.S.A. Second, we investigated the reproductive system of this species in its introduced range, assessing the number of queens per nest, the number of matings per queen, the possibility that queens reproduced through thelytokous parthenogenesis, and the relatedness among nestmate workers. A comparison of colony genetic structure in the native and introduced ranges allowed us to determine whether the recent introduction of *N. fulva* induced a shift in its social system, from multicoloniality to unicoloniality. Lastly, we performed behavioral assays testing whether workers from different colonies within the invasive range recognize each other as colony mates in order to define the number and the extent of the supercolonies observed in the U.S.A.

## Results

**Fig. 1:**
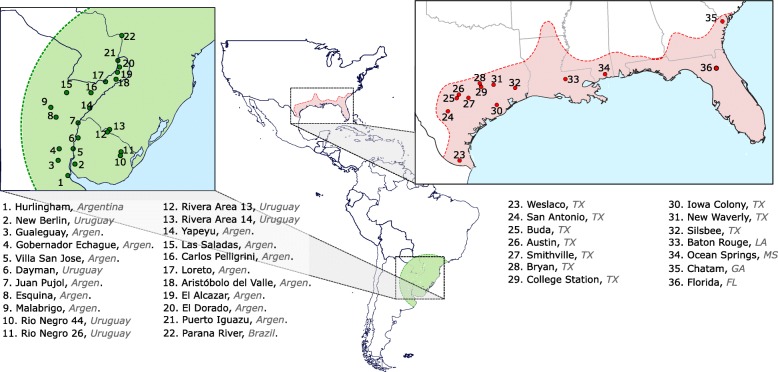
Sampling map of the tawny crazy ant in its native and introduced ranges

All thirteen microsatellite loci developed and used in this study were polymorphic in the native range of *N. fulva* with allele numbers ranging from 5 to 21 (X ± SD = 11.7 ± 3.9). In the introduced range, all microsatellite markers were polymorphic with a single exception (L12). Allele numbers at polymorphic loci ranged from 3 to 8 (X ± SD = 4.8 ± 2.0). Allele diversity in the U.S.A. was significantly lower than that observed in the native range (Wilcoxon test *P* < 0.01): 153 alleles out of a total of 156 alleles were found in South American populations, while 61 alleles were found in U.S.A. populations (Fig. 1).

A total of nine mitochondrial haplotypes were found. All haplotypes were found in the native range, while only two were uncovered in the introduced range. In the native range, the mean genetic distance within group was 0.034 (20.04 bp difference on average), but only 0.002 (1.45 bp difference in average) in the introduced range.

### Population and colony structure

**Fig. 2:**
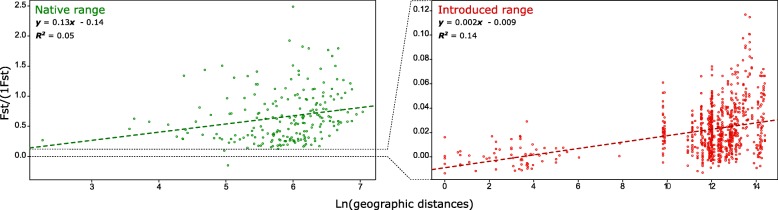
Correlations between genetic differentiation between nests and geographic distances (isolation by distance) of the tawny crazy ant in its native and introduced ranges using microsatellite markers

**Fig. 3:**
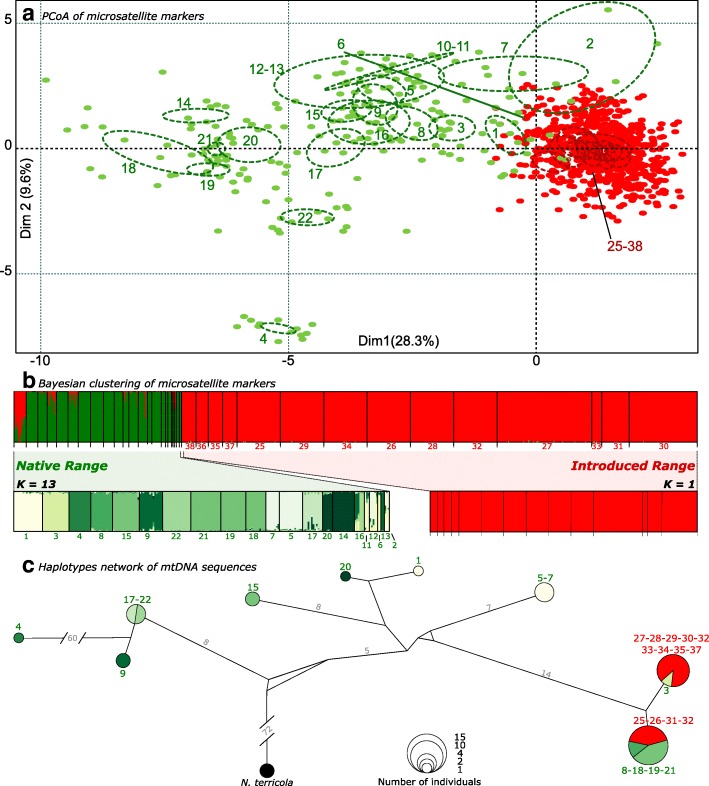
**a** Principal Components Analysis of the microsatellite markers for all the populations of *N. fulva* sampled. **b** Graphical representation of STRUCTURE results for different values of K genetic groups using the entire dataset (*n* = 937; *N* = 63 nests). Simulation using a single individual per nest gives similar results (Additional file 1: Figure S1). Each group is characterized by a color; and each individual is represented by a vertical bar according to its probability to belong to each group. A different simulation was run for our overall sampling and then for both native and introduced ranges separately. **c** Haplotypes network for the COI mitochondrial marker of *N. fulva* in its native and introduced populations. Circle sizes are proportional to the number of sequences observed in the dataset and branch lengths indicate the number of mutations between haplotypes. *N. terricola* is used as an outgroup

Significant population structure was found in the native range of *N. fulva*, with 11.3% of the total genetic diversity distributed among the different localities. We also observed significant differentiation among nests within localities (mean *F*_ST_ ± SD = 0.36 ± 0.14); this level accounted for 25.9% of the variation (AMOVA, Table 1). We found a positive relationship between pairwise *F*_ST_ and geographical distance (Fig. 2). Genetic clustering in the native range was also evident using Principal Component Analysis, as the different localities scattered along the first principal component (Fig. 3a). STRUCTURE analyses including only ants from the native range revealed 13 genetic groups (optimal k = 13). However, native populations clustered into a single genetic group when all samples from native and introduced ranges were analyzed (Fig. 3b, Additional file 1: Figure S1).

**Table 1:** Analysis of molecular variance (AMOVA) at different hierarchical levels for both native (*Nat*.) and introduced (*Int*.) populations of *N. fulva*

Source of variation	Sum of squares	Variance Components	Percentage variation
Among localities			
Nat.	549,18	0,45	11,31
Int.	125,93	0,068	2019
Among nests within localities			
Nat.	89,34	1,04	25,95
Int.	96,07	−0,005	−0,144
Within nests			
Nat.	1031,43	2,51	62,74
Int.	4446,94	3351	98,125
TOTAL			
Nat.	1669,95	4,01	
Int.	4658,94	3,41

**Fig. 4:**
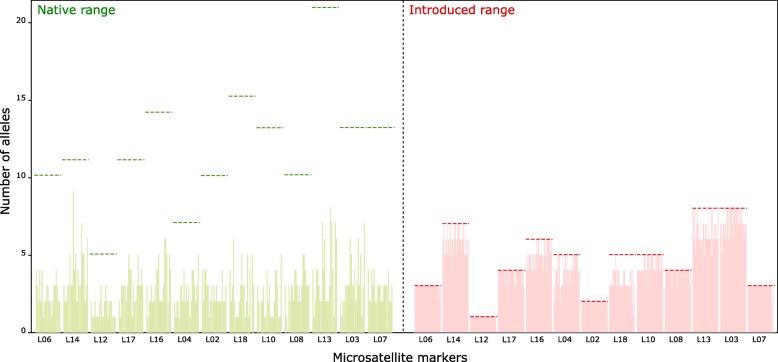
Number of alleles for each of the 13 microsatellite markers in the native and introduced ranges of *N. fulva*. Horizontal dotted lines represent the overall number of alleles for both the native and introduced populations, while the vertical bars represent the number of alleles uncovered within each of the 22 nests in the native range and the 14 nests in the introduced range

Population structure among introduced U.S.A. populations was quite low, in contrast to the results uncovered in the native range. Differentiation among localities accounts for only 2.0% of the genetic diversity and 98.1% of variation is due to variation within nests, indicating each individual nest contains nearly as much diversity as found in the entire introduced range (Fig. 4). No genetic diversity (− 0.144%) was distributed among nests within localities. The mean *F*_ST_ between nests was close to zero (*F*_ST_ ± SD = 0.021 ± 0.019) and *G*-tests revealed that most of the nests sampled in the introduced range could not be differentiated; including those separated by several thousands of kilometers (*all P* were non-significant after Bonferroni correction*s*). Nonetheless, a positive relationship between pairwise *F*_ST_ and geographic distance was found in the introduced range, but the scale of differentiation was considerably lower than in the native range (Fig. 2). The absence of genetic structure in introduced populations was supported by STRUCTURE analyses, which suggests all individuals in the U.S.A. belong to a single cluster (k = 1, regardless of whether or not individuals from the native range were included in the analyses; Fig. 3b, Additional file 1: Figure S1). The lack of genetic structure was also found using PCoA, as all introduced populations densely clustered together without any discernible differentiation along the axes (Fig. 3a). All together, these findings clearly suggest that all nests sampled across the U.S.A. populations of *N. fulva* form a single supercolony.

### Reproductive system and genetic relatedness

Colonies of *N. fulva* in the introduced range were spatially expansive, making a complete excavation and a precise count of queen number impossible. Despite this, 0 to 20 queens were typically found in most of the nests sampled, with up to 300 queens discovered from a single nest. Moreover, the assignment of worker genotypes was not compatible with the occurrence of a single queen for any of the nests sampled. This suggests that multiple queens shared reproduction and/or that workers freely moved between nests in the U.S.A.

**Fig. 5:**
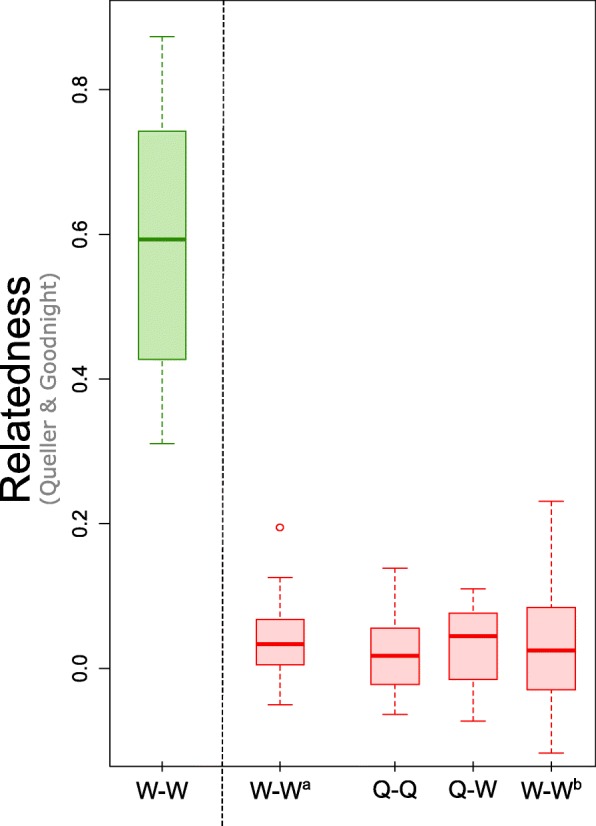
Overall relatedness coefficients among nestmate workers in the native (left, r_W-W_) and introduced (right, r_W-W_^a^) ranges of *N. fulva*. Relatedness coefficients uncovered among queens (r_Q-Q_), between queens and workers (r_Q-W_) and among nestmate workers (r_W-W_^b^) for the extensive sampling of 16 nests in the introduced range

**Fig. 6:**
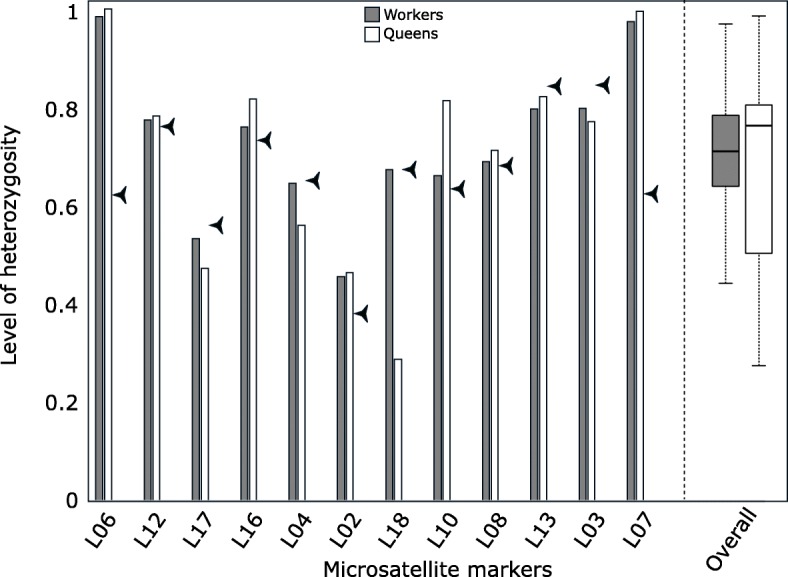
Level of heterozygosity in worker (grey) and queen (white) castes for each microsatellite marker and the overall microsatellite dataset. Arrows indicate the level of heterozygosity expected in the population

A total of 67 mother-queens and 60 winged-queens from extensive sampling in 16 nests from four localities were genotyped to determine whether new queens of this species are produced through thelytokous parthenogenesis. The relatedness among queens within nests was close to zero (*r*_q-q_ ± SD = 0.02 ± 0.06) and not significantly different from the relatedness among workers within the same nests (*r*_w-w_^b^ ± SD = 0.04 ± 0.09; Fig. 5). Moreover, the levels of heterozygosity did not differ between worker and queen castes (Wilcoxon test, *P* = 0.968), indicating that both castes are produced through classic sexual reproduction (Fig. 6).

A total of seven queens were successfully isolated with a group of workers into subcolonies and produced enough progeny to reliably infer whether polyandry occurs through mother-offspring comparisons. All the genotypes of worker pupae were unequivocally assigned to the genotype of the putative mother queen, and were consistent with a single mating of the queen in all seven subcolonies analyzed. Despite the low level of genetic diversity in the introduced range, the probability of non-detection of two males as a result of carrying the same alleles at all loci is very low (*P* non-detection = 2.99 × 10^− 6^).

The above findings indicate that introduced populations of *N. fulva* have highly polygynous nests containing up to hundreds of queens, each of them being singly mated. As a result, together with its supercolonial structure, the mean relatedness among nestmate workers is close to zero in the introduced range (*r*_w-w_^a^ ± SD = 0.04 ± 0.05). This finding is in sharp contrast with the high relatedness among nestmates observed in the native range (*r*_w-w_ ± SD = 0.57 ± 0.19) (Fig. 5). Interestingly, the relatedness in the introduced range differs from zero (*r*_w-w_ = 0.16) when the global population is taken as a reference.

### Behavioral assays

**Fig. 7:**
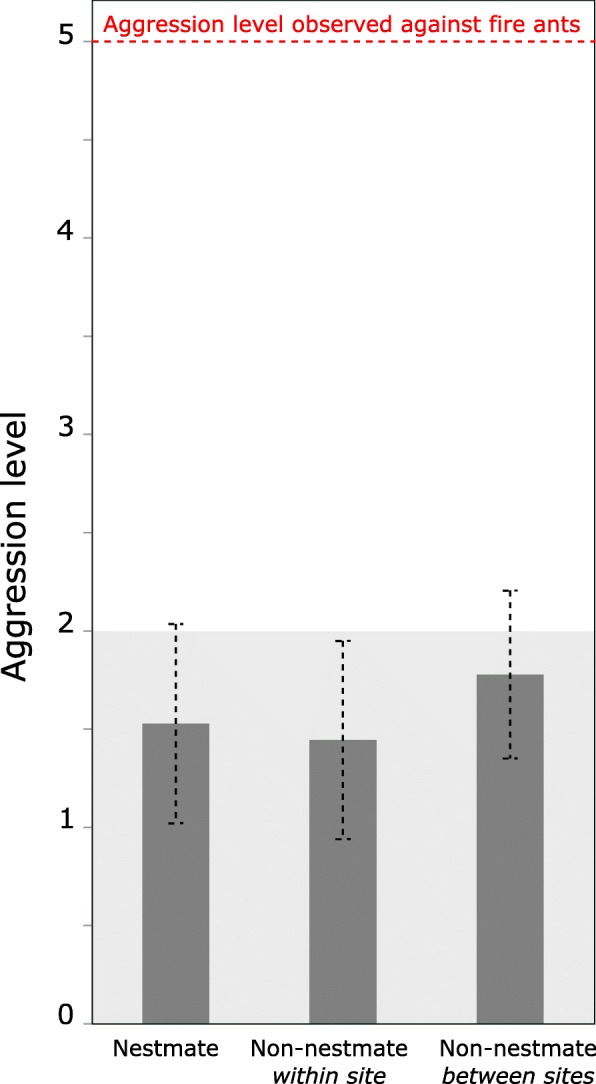
Aggression level between workers of *N. fulva* from different origins: nestmate, non-nestmate from the same site, and non-nestmate from different sites. Grey zone indicates non-aggressive behaviors (a score from 0 to 2); the red dotted line indicates the maximum level of aggression, uncovered during all the assays against the heterospecific fire ant *Solenopsis invicta*

In aggression assays of *N. fulva*, including nestmates, non-nestmates from the same locality, and non-nestmates from different localities, aggressive behaviors were not observed, with all assays obtaining a score of 1 (still or huddling) and 2 (antennation, allogrooming or trophallaxis) (Fig. 7). Moreover, no significant difference in the level of aggression was observed between *N. fulva* workers from the same nest, the same locality or different localities (Kruskal-Wallis test, *P* = 0.07). The number of trophallactic events was too low to test for a possible difference in the sharing of food among workers, regarding whether they belong to the same nest, the same locality or different localities. Aggressive behavior was observed when *N. fulva* was confronted with *S. invicta*, where in all assays the maximum level of aggression was recorded, revealing that *N. fulva* workers were fully capable of acting in an aggressive manner.

## Discussion

Our large-scale genetic and behavioral analyses of the invasive tawny crazy ant provide several new insights into the biological invasion and social system of this ant in its native and invasive (U.S.A.) ranges. Genetic data suggest this ant species experienced a genetic bottleneck following its introduction in the U.S.A. that led to a significant reduction (60%) in genetic diversity. Population genetic analyses show that *N. fulva* exhibits a multicolonial organization in its native range, with colonies genetically distinct from each another. In contrast, we found that this species displays a unicolonial system with no clear boundaries between nests in its invasive range. This latter finding is supported by the lack of genetic differentiation among nests within populations as well as between geographic populations, and relatedness coefficients among nestmate workers close to zero in introduced populations. Each invasive nest was headed by several, up to hundreds of singly-mated reproductive queens. Behavioral tests reveal no aggressive behaviors toward conspecifics from different nests, even ones separated by several hundred kilometers. Overall, these results suggest that the entire U.S.A. range of the species forms a single large supercolony spreading more than 2000 km.

### Population bottleneck and inbreeding

In our study, we uncovered a loss of genetic diversity between native and introduced populations. Such reduction may be particularly costly for hymenopteran species because of their sex determination system. In these species, the sex of an individual is controlled by a single complementary sex-determining locus (multi-locus CSD is known but rare [47]). Heterozygous individuals at this locus develop into females while homozygous individuals develop into males. Females are diploid heterozygous individuals usually produced by sexual reproduction, whereas males arise from unfertilized eggs through arrhenotokous parthenogenesis, and are therefore haploid (i.e., thus homozygous) individuals [48]. However, diploid individuals, homozygous at this locus, which can result from mating between individuals carrying the same sex allele (matched mating), develop as diploid males. Production of diploid males represents a cost for colonies because they are effectively sterile in most hymenopteran species [48–51]. Loss of allelic diversity at the sex locus as a result of a population bottleneck significantly increases the chances of matched matings [52]. As one example, in the introduced populations of the red fire ant *Solenopsis invicta,* colonies produce a higher proportion of diploid males than those from native populations [53, 54].

Some ant species have however evolved unorthodox reproductive modes, which may facilitate invasiveness by acting as pre-adaptations against the genetic loss due to bottlenecks during invasions [55]. In some populations of four invasive ant species, *Wasmannia auropunctata, Vollenhovia emeryi, Anoplolepis gracilipes* and *Paratrechina longicornis*, queens are clones of their mothers and males are clones of their fathers, whereas workers arise from classical sexual reproduction [35, 55–57]. Male and female gene pools are completely segregated, even those produced by the same mother queen [58, 59]. In these species, a single-mated queen may thus establish an introduced population, producing 100% heterozygous workers. This queen may also produce new queens and males able to mate together inside the nest; yet still maintain heterozygosity in their worker offspring. Clonality was recently recorded in the native range of *W. auropunctata* in southern South America [60, 61]. This strategy thus circumvents the costs of inbreeding after an introduction event over an unlimited number of generations [55, 59] and act as a pre-adaptive trait to invasion. In our study, the level of heterozygosity is not significantly different between workers and queens of *N. fulva,* indicating that they are both produced through classic sexual reproduction.

Formation of supercolonies seems to be a common trait of invasive social insects [22, 62], allowing a rapid and efficient monopolization of resources to achieve local dominance, mainly in the introduced range where the competitive pressure exerted by the members of local ant community is lower than in the native range [2, 8, 63, 64]. Supercolonies have been reported numerous times in various invasive ant species, such as *Linepithema humile* [9], *Monomorium pharaonis* [65], *Pheidole megacephala* [32], *Anoplolepis gracilipes* [66], and *Lasius neglectus* [67], and has also been reported in invasive populations of the termite *Reticulitermes urbis* [68]. Interestingly, the sizes of supercolonies apparently varies considerably among species, and occasionally even within species. For example, the invasive big-headed ant, *P. megacephala*, forms a single large supercolony covering up to 3000 km across northeastern Australia [32]. In contrast, the yellow crazy ant, *A. gracilipes,* inhabiting a small geographic area in northeastern Borneo, comprises at least six supercolonies [66]. In *L. humile*, two supercolonies are reported in the invasive range in southern Europe; one supercolony is 6000 km long, while the other is only a few km long [9]. In this same species, the invasive area of California comprises at least five supercolonies ranging in areas from 1 to 1000 km [69], while four supercolonies were uncovered in Japan [27, 70], and several in the southeastern U.S.A. [71]. Overall, these results suggest supercoloniality is a common trait in social insects, but the number and the size of their supercolonies can differ greatly among and within species.

Despite the lack of aggressiveness within supercolonies, genetic identities of two adjoining supercolonies can be maintained because workers display strong aggression towards allocolonial sexuals and workers at colony boundaries, as observed in the Argentine ant [69, 72, 73]. This aggression towards allocolonial sexuals strongly reduces potential for mating between partners from distinct supercolonies. Thus, gene flow is reduced between abutting supercolonies, resulting in maintenance of genetic distinctiveness even after prolonged contact with one another [9, 21, 69, 74]. Supercolony differentiation has been suggested to come about one of two ways. On the one hand, supercolonial structure may stem from an initial colony differentiation, in which different supercolonies came from multiple introductions. These distinct introductions from genetically and chemically differentiated source populations are more likely to result in distinct supercolonies in the invasive range [75, 76]. For example, the worldwide supercolonies of the Argentine ant originated from at least seven founding events out of the native area in Argentina [75]. The dominant supercolonies of Europe, Japan and California probably arose from the same primary introduction [75] and consist of a single supercolony that globally expanded through secondary introductions, since these populations are not aggressive toward each other [76] and have similar hydrocarbon profiles [77]. On the other hand, supercolony differentiation may occur through divergence after introduction. Queen recruitment, intranidal mating and female dispersal through budding may lead to a reduction of gene flow between geographically separated fragments of the same initial supercolony. Over time, the accumulation of genetic and cuticular hydrocarbon (CHC) differentiation may result in mutual aggression between fragments [78]. In the introduced population of *L. humile* in Corsica, the clear reduction of gene flow between the island and the mainland supercolonies has led to noticeable chemical and behavioral differentiation [79]. A similar pattern has been reported in a single population in the Californian invasive range, yet coming from the introduction event that gave rise to the other supercolonies [80], and in *A. gracilipes* in Borneo, in which spatial separation has enhanced genetic and CHC differentiation over time [78]. Eventually, these cases may result in allopatric fragmentation if enough differentiation occurs before both fragments come into contact again. However, no pattern of isolation by distance has been found within supercolonies of other invasive ant species [9, 34, 71, 74], suggesting that gene flow is high enough in these supercolonies to prevent differentiation among geographically distant areas within supercolonies.

In *N. fulva*, no abrupt genetic transition was discovered across all introduced *N. fulva* populations studied, suggesting that this species forms a single large supercolony from Texas to Florida. We uncovered a weak pattern of isolation by distance in the introduced range, several orders of magnitude lower than that in the native range. This result may stem from the absence of nuptial flights in *N. fulva* and its invasion front expansion through budding. These features usually lead to a genetic population viscosity, which may, over time, result in population differentiation. The invasion of *N. fulva* in the U.S.A. is recent, and may not have had sufficient time to induce genetic differentiation between localities and split the invasive range into distinct supercolonies. Although we cannot exclude that other supercolonies of *N. fulva* are present but were not sampled, our results suggest that introduced populations in the U.S.A. may come from a single introduction from South America, which then spread through human mediated jump dispersal. The hypothesis of a single introduction is also supported by the positive relatedness (*r*_w-w_ = 0.16) observed when the global population is taken as reference.

Unicoloniality results in several ecological advantages in terms of colony growth, nest density, productivity and survival, and may favor invasive success outcompeting native species through resource monopolization [2]. But on the other side of the coin, unicoloniality reduces to zero the relatedness between nestmate workers, and, thus, the workers’ indirect benefits from helping. In this context, selfish behaviors are expected to disrupt social cohesion within colonies [81, 82]. For this reason, unicoloniality might represent an evolutionary dead-end; an idea supported by the fact that there is no unicolonial species but only unicolonial populations, and by its scattered distribution along the ant phylogeny [22]. In *N. fulva*, the relatedness between nestmate workers did not differ from zero in the introduced range, while it varied from 0.29 to 0.86 in the native range. A similar loss of relatedness has also been reported in several supercolonial populations of the species *L. humile*, *L. neglectus*, *P. megacephala*, and *S. invicta* [2, 32, 83, 84]. However, most of these species were comprised of several genetically distinct supercolonies, with members of the same colony more related to each other than to members of other supercolonies. In this case, it is important to measure relatedness with respect to the local competing population rather than to the global population [22]. Taking the two supercolonies of *L. humile* in the Southern European range as an example, it is unlikely that two workers separated by thousands of kilometers still compete with each other. Therefore, in most parts of the supercolony, workers most likely compete with colonymates; while selection for altruism should only take place at colony boundaries [22]. In our study, the whole introduced range seems to comprise a single supercolony, even if workers within the introduced range are more related to each other than to workers from native populations, introduced workers do not compete with native workers, making introduced relatedness equivalent to zero. In contrast, several supercolonies of *A. gracilipes* inhabit the island of Borneo [66]. In this limited area, workers of a given supercolony are more likely to compete against workers from other supercolonies. The relatedness coefficients observed in this species are quite high, making social cohesion sustainable when supercolony size is reduced [35]. Actually, supercolonies of smaller size are uncovered in native populations of the Argentine ant [9, 21, 34, 85, 86] and the little fire ant [61, 87, 88]. In noninvasive species, the turnover of supercolonies suggests the occurrence of local competition [89]. Overall, these outcomes suggest that unicoloniality is not only a derived trait in invasive populations, but might represent a sustainable social strategy when the reduction of relatedness outweighs its ecological advantages.

## Conclusions

Overall, this study shows that like several unrelated ant species, introduced populations of *N. fulva* developed a unicolonial organization, giving another example of the independent evolution of this social structure in ants [22]. Yet, the scattered distribution of unicoloniality along the ant phylogeny casts doubt on the long-term stability of this system, in which one might expect a complete breakdown of co-operation due to the absence of relatedness among nestmates. This study reports another ant species exhibiting plasticity in reproductive strategy and behavior that allows it to take advantage of the loss of genetic diversity in the invasive range. Further studies investigating whether native populations of this species consist of small localized colonies or smaller supercolonies should shed further light on whether the large supercolonies formed in the U.S.A. are due to a post-introduction shift in social structure or whether it is related to pre-adapted traits present in the native population.

## Methods

A total of 36 populations of *N. fulva* were mainly collected between 2015 and 2017 (Fig. 1). Sampling comprised of 14 populations in its introduced range from Texas to Georgia, U.S.A. and 22 populations in its native range in South America (Additional file 2: Table S1). For each population, 1 to 8 nests were sampled (X ± SD = 1.7 ± 1.6; *N* = 65). Colonies from Texas populations were brought back alive to the laboratory, where they were maintained under standard conditions (28 ± 2 °C, 12:12 h photoperiod, and fed sugar water and cockroaches) for behavioral and breeding system analyses. A subset of individuals from the Texas colonies were then removed and stored in 95% ethanol. All samples from other U.S.A. localities and from South America were immediately stored in 95% ethanol for subsequent genetic analyses.

### Genetic procedures

For each individual, total genomic DNA was extracted following a modified Gentra Puregene extraction method (Gentra Systems, Inc. Minneapolis, MN, USA). Thirteen new microsatellite markers (Additional file 3: Table S2) were developed for *N. fulva* based on the transcriptome generated by Valles et al. (2012; [90]). Amplicons were labelled with 6-FAM, VIC, PET or NED dye to facilitate multiplexing. PCR conditions and multiplexing arrangements are given in the online supplementary material (Additional file 3: Table S2). PCR were run on a Bio-Rad thermocycler T100 (Bio-Rad, Pleasanton, CA, U.S.A.). PCR products were sized against LIZ500 internal standard on an ABI 3500 capillary sequencer (Applied Biosystems, Foster City, CA, U.S.A.). Allele scoring was performed using Geneious v.9.1 [91]. A fragment of the COI mitochondrial gene was also sequenced using the *Jerry* and *Pat* primer pair previously developed for *Apis mellifera* [92]. PCR products were purified with EXOSAP-it PCR purification kit (Affymetrix), and sequenced using the ABI BigDye Terminator v.3.1 Cycle Sequencing Kit on an ABI 3500 Genetic Analyzer (Applied Biosystems). Base calling and sequence reconciliation were performed using CodonCode Aligner (CodonCode Corporation, Dedham, MA, U.S.A.).

### Population and colony structure

For the mitochondrial dataset, we sequenced 1–3 workers for each population, which overall included 26 workers in 14 native locations and 14 workers in 13 introduced locations in the U.S.A. We included two samples of *Nylanderia terricola* from one location in Texas as an outgroup. The conservation of some samples in alcohol was not optimal, especially those from the native range, often resulting in poor quality DNA, and some samples could not be sequenced successfully. Haplotype network was used to visualize phylogeographic relationships between mitochondrial haplotypes. Networks were produced by the median-joining method [93] implemented in the program NETWORK v.4.6.1.1 (available at http://www.fluxus-engineering.com/). Nucleotide diversity and genetic distance were compared within and between populations using MEGA v. 5.0 [94].

For microsatellite analyses, 2–20 individuals per nest were genotyped at 13 microsatellite markers in each locality (X ± SD = 15.11 ± 5.22; *n* = 937; *N* = 63 nests). The number of alleles, allele frequencies, measures of observed and expected heterozygosity, and *F*-statistics were determined using FSTAT [95]. We looked for evidence of a recent bottleneck by testing for an excess of heterozygotes with Bottleneck 1.2 [96]. The loss of rare alleles after a bottleneck is expected to lead to excess heterozygosity compared with expectations under mutation-drift equilibrium [96]. We used the two-phase model (TMP) to generate expected heterozygosity in Bottleneck 1.2.

For both native and introduced ranges, the hierarchical partitioning of the genetic diversity among localities, among nests within localities, and within nests was assessed using analysis of molecular variance (AMOVA) implemented in Arlequin [97]. We assessed the level of genetic differentiation between localities by estimating genetic differentiation *F*_ST_, and tested its statistical significance by a permutations test using FSTAT [95]. We investigated population structure and isolation-by-distance by plotting [*F*_ST_/(1 – *F*_ST_)] coefficients between pairs of nests against the *ln* of their geographical distance (Slatkin 1993). The significance of the correlation was tested using Mantel tests implemented in GENEPOP ON THE WEB [98]. We visualized population structure by plotting individuals on a Principal Component Analysis (PCoA) using *FactoMineR R* package [99]. We tested for the presence of genetic structure within and among populations inferring the number of genetic clusters (K) in our samples using the Bayesian clustering method implemented in STRUCTURE v.2.3 [100]. Simulations were run separately for all individuals with K ranging from 1 to 36, for individuals from the native range only (K from 1 to 22), and for individuals from the introduced range only (K from 1 to 14). The simulations were replicated 10 times for each number of K. The analyses were run using a combination of a correlated-allele frequencies and an admixture model. Each run comprised a first step of a 5 × 10^4^ burn-in period and 1 × 10^5^ iterations of the MCMC. The log-likelihood value and the ΔK method [101] implemented in Structure Harvester v.0.6.8 [102] were used to estimate the most likely number of clusters. Finally, whether different nests within populations belonged to the same colony was determined by comparing genotypic frequencies at all loci with a log-likelihood (G)-based test of differentiation using GENEPOP ON THE WEB [98]. The overall significance across loci was determined using a Fisher’s combined probability test after Bonferroni correction for multiple comparisons (α after Bonferroni correction = 0.00006).

### Reproductive system and genetic relatedness

We estimated the number of queens per nest, the number of matings per queen, genetic relatedness among nestmate workers, and possible production of queens through thelytokous parthenogenesis using samples from the introduced range. The presence of multiple queens per nest was assessed directly from field observations, and polygyny was confirmed genetically when all the worker genotypes from a nest could not be assigned unambiguously to a single queen. We estimated queen mating frequency by establishing artificial subcolonies containing a single queen and ~ 100 workers using Texas colonies. Care was taken to remove all brood to ensure that all the new workers produced in a subcolony were the offspring of the introduced queen. Each subcolony was kept under standard rearing conditions over a three month period or until the queen produced at least eight worker pupae. All mother queens and their newly produced pupae (X ± SD = 10.0 ± 2.6) were genotyped at all 13 microsatellite loci at the end of the experiment. The number of matings per queen was inferred by reconstructing parental genotypes from mother-offspring inferences using the maximum likelihood algorithm implemented in COLONY 1.2 program [103]. As genetic diversity was low in the introduced range (see Results), we calculated the probability of non-detection of a second male carrying the exact same genotype at all loci studied using Boomsma and Ratnieks (1996; [104]) equation:

$$ \boldsymbol{P} non- detection={\prod}_j{\sum}_i{f_{ij}}^2 $$

where *f*_*ij*_ is the frequency of the allele *i* at the locus *j.*

Relatedness coefficients (r) among nests were estimated using COANCESTRY v.1.0 [105], following the algorithm described by Queller & Goodnight (1989; [106]). Relatedness coefficients were calculated separately for the introduced and the native range to account for the differences in allele frequencies between populations. We also calculated relatedness within the introduced range using the global population as a reference for allelic diversity. Finally, we assessed the possibility that queens produce new queens via thelytokous parthenogenesis by comparing heterozygosity level and relatedness between castes in the introduced range using an extensive sampling of 16 nests from four localities in Texas (27, 30, 31 and 33). Thelytokous parthenogenesis through automixis decreases homozygosity over time [107, 108]. Parthenogenetic production of queens would lead to a difference in observed heterozygosity between queen and worker castes due to a decline of heterozygosity and increased relatedness among queens compared to the sexually produced workers.

### Behavioral assays

Within a week of collection, standardized aggression tests were conducted by placing two workers in a 5 cm petri dish arena for 5 min. Workers were not starved before the beginning of the experiment. The arena floor was covered with filter paper to prevent odor transfer between replicates, and the sides were coated with Fluon to prevent escapes. Interactions were scored on a 5-level scale: levels 1 (ants still or huddled together) and 2 (antennation, allogrooming or trophallaxis) were considered non-aggressive behaviors, whereas levels 3 (biting and quickly releasing), 4 (prolonged biting > 3 s) and 5 (balling, fighting, spraying formic acid) were considered as agonistic. Aggression tests were conducted with three nests per locality for four localities separated by at least 30 m in the introduced range in Texas (localities 26, 27, 28 and 29; Fig. 1). Interactions were measured between nestmates, then between non-nestmates, either from the same or different localities. We also tested the interactions between one worker from each locality and a red imported fire ant worker (*Solenopsis invicta*), to control for the ability of *N. fulva* to be aggressive. Each combination was replicated three times, yielding a total of 36 encounter type assays between nestmates, 36 between non-nestmates from the same locality, 18 between non-nestmates from a different locality, and 12 against a fire ant. Aggression levels were compared using ANOVA tests between groups using R software [109]. All figures were made using the free software Inkscape v.0.92 (available at http://www.inkscape.org/).

## Additional files

Additional file 1: **Figure S1.** Graphical representation of STRUCTURE results for different values of K genetic groups. (PDF 33 kb)

Additional file 2: **Table S1.** List of sample names with information on localities and accession numbers. (PDF 38 kb)

Additional file 3: **Table S2.** Primer sequences, PCR optimization and multiplexing for each of the markers used in our study. This also includes the methods used to estimate detection of null alleles and linkage disequilibrium for the microsatellite markers analyses. (PDF 48 kb)

## Abbreviations

CHC: Cuticular hydrocarbon
COI: Cytochrome oxidase 1
CSD: Complementary sex-determining (locus)
