# Supplementary material for: Supercolonial structure of invasive populations of the tawny crazy ant Nylanderia fulva in the US
Source: BMC Evol Biol. 2018 Dec 29;18:209. doi: 10.1186/s12862-018-1336-5 (PMC6310932; doi:10.1186/s12862-018-1336-5)
Supplement: Supplementary file 1 — Figure S1. Graphical representation of STRUCTURE results for different values of K genetic groups. (PDF 33 kb) [file 12862_2018_1336_MOESM1_ESM.pdf]

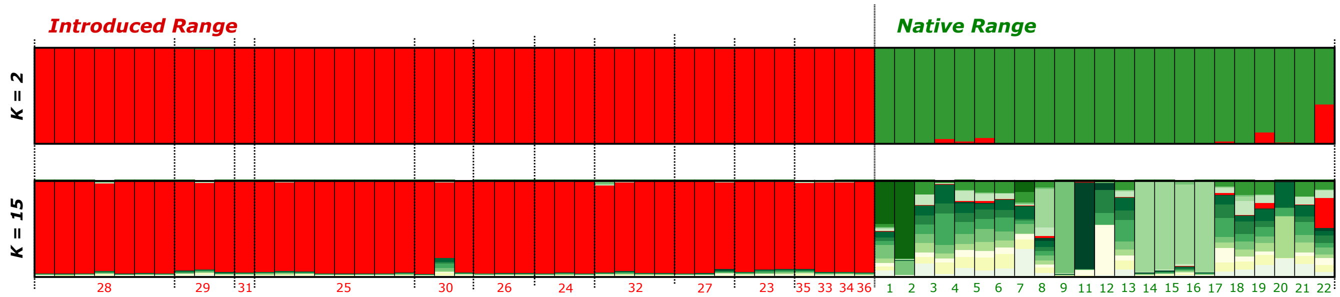

Figure S1: Graphical representation of STRUCTURE results for different values of K genetic groups. Each group is characterized by a color, and each individual is represented by a vertical bar according to its probability to belong to each group. The simulation was run for our overall sampling, with a single individual per colony. The most likely number of genetic groups in the population is (K = 2), while (K = 15) is the second most likely, according to Evanno et al. (2005) method.
