# Supplementary material for: Supercolonial structure of invasive populations of the tawny crazy ant Nylanderia fulva in the US
Source: BMC Evol Biol. 2018 Dec 29;18:209. doi: 10.1186/s12862-018-1336-5 (PMC6310932; doi:10.1186/s12862-018-1336-5)
Supplement: Supplementary file 2 — Table S1. List of sample names with information on localities and accession numbers. (PDF 38 kb) [file 12862_2018_1336_MOESM2_ESM.pdf]

Supercolonial structure of invasive populations of the tawny crazy ant *Nylanderia fulva* in the US

Eyer et al. (Table S1)

Range	Location	nest	workers	Queens	Gynes
Introduced	Bryan	NF1.1	4	3	8
Introduced	Bryan	NF1.2	4	0	8
Introduced	Bryan	NF1.3	4	0	8
Introduced	Bryan	NF1.4	4	0	8
Introduced	Bryan	NF1.5	4	0	8
Introduced	Bryan	NF1.6	4	0	8
Introduced	Bryan	NF1.7	4	5	8
Introduced	CollegeStation	NF2.1	8	6	0
Introduced	CollegeStation	NF2.2	8	4	0
Introduced	CollegeStation	NF2.3	8	2	4
Introduced	NewWaverly	NF3.4	4	8	0
Introduced	Buda	NF4.1	6	10	0
Introduced	Buda	NF4.2	6	4	0
Introduced	Buda	NF4.3	6	9	0
Introduced	Buda	NF4.5	6	8	0
Introduced	Buda	NF4.6	6	10	0
Introduced	Buda	BU01	20	0	0
Introduced	Buda	BU02	20	0	0
Introduced	Buda	BU03	20	0	0
Introduced	IowaColony	IC01	20	0	0
Introduced	IowaColony	IC02	20	0	0
Introduced	IowaColony	IC03	20	0	0
Introduced	Austin	AU01	20	0	0
Introduced	Austin	AU02	20	0	0
Introduced	Austin	AU03	20	0	0
Introduced	SanAntonio	SA01	20	0	0
Introduced	SanAntonio	SA02	20	0	0
Introduced	SanAntonio	SA03	20	0	0
Introduced	Silsbee	SI01	20	0	0
Introduced	Silsbee	SI02	20	0	0
Introduced	Silsbee	SI03	20	0	0
Introduced	Smithville	SM01	20	0	0
Introduced	Smithville	SM02	20	0	0
Introduced	Smithville	SM03	20	0	0
Introduced	Weslaco	WE01	20	0	0
Introduced	Weslaco	WE02	20	0	0
Introduced	Weslaco	WE03	20	0	0
Introduced	Chatam	Geo01	20	0	0
Introduced	BatonRouge	Lou01	20	0	0
Introduced	OceanSprings	Miss01	17	0	0
Introduced	Florida	Florida	20	0	0
Native	NewBerlin		3	0	0
Native	RiveraArea 13		2	0	0

Native	RiveraArea 14	5	0	0
Native	Dayman	2	0	0
Native	RioNegro	3	0	0
Native	CarlosPellegrini	6	0	0
Native	Yapeyu	10	3	0
Native	ElDorado	5	1	0
Native	Loreto	10	2	0
Native	SanJose	11	3	0
Native	JuanPujol	7	1	0
Native	Aristóbulo	5	2	5
Native	ElAlcazar	15	0	0
Native	PuertoIguazu	18	0	0
Native	ParanaRiver	16	0	1
Native	Malabrigo	14	0	0
Native	LasSaladas	16	0	0
Native	Esquina	13	0	0
Native	Gobernador	11	0	2
Native	Gualeguay	14	2	0
Native	Hurlingham	17	0	0
		Average	15,11	
		SD	5,22	
		Total	937	
