# Supplementary material for: Supercolonial structure of invasive populations of the tawny crazy ant Nylanderia fulva in the US
Source: BMC Evol Biol. 2018 Dec 29;18:209. doi: 10.1186/s12862-018-1336-5 (PMC6310932; doi:10.1186/s12862-018-1336-5)
Supplement: Supplementary file 3 — Table S2. Primer sequences, PCR optimization and multiplexing for each of the markers used in our study. This also includes the methods used to estimate detection of null alleles and linkage disequilibrium for the microsatellite markers analyses. (PDF 48 kb) [file 12862_2018_1336_MOESM3_ESM.pdf]

Supercolonial structure of invasive populations of the tawny crazy ant *Nylanderia fulva* in the US
Eyer et al. (Table S2)

Primer	Dye	Sequence (5'-3')	Mix	Amount (µl)
L06 - F	FAM	CCTATACTCCTATCCTCCCATCG	1,1	0.24
L06 - R		GCTTCTTGAAGTAGCAGCTAGAGGAGG		0.24
L14 - F	FAM	GCTGGTGTGTATCGATCCCTC		0.2
L14 - R		GCTTCTATAACTGGATTCTCTTGTCGGC		0.2
L12 - F	FAM	TCTCTCAAAGCATCCTCAGAAC		0.2
L12 - R		GCTTCTCCAGGTGATAGATGAGCATGC		0.2
L04 - F	NED	GATGTGAGATACAAGGTCGGAG	1,2	0.16
L04 - R		GCTTCTCTATTACCACTCGATCGTCACG		0.16
L02 - F	PET	CGTAATCGCGACTAGGTTAGAG		0.2
L02 - R		GCTTCTCAACTGTCATTGATGTGCCAAG		0.2
L18 - F	PET	GAGTAGGTACGTGAAAGAGGAC		0.24
L18 - R		GCTTCTCGATAAAGCTACACCGTCTCTC		0.24
L17 - F	VIC	GAAGTGGATGGAACGAGGAATC	1,3	0.16
L17 - R		GCTTCTCATATATATGTTTGCAAGCGAGC		0.16
L16 - F	VIC	GTGAATCCTCGATACTTGGCTG		0.24
L16 - R		GCTTCTGAGGAAGAGGTCGAAGGAGTC		0.24
L10 - F	FAM	GAATACGTCGAGACTTACTGGC	2,1	0.2
L10 - R		GCTTCTTTTGTCTGTCTGCCTGCTTATC		0.2
L08 - F	FAM	TCTCTCTCTGTTCCGCAAATTC		0.2
L08 - R		GCTTCTAGATCGAATTCAATGCACAATC		0.2
L13 - F	VIC	CCGCAATTACATGGCTTTGAAC		0.45
L13 - R		GCTTCTTAGATACAGGACGTTACACAGC		0.45
L03 - F	NED	AAGTTTCCTTAATATCCCGCGG	2,2	0.25
L03 - R		GCTTCTTATACGGTGCCTTAACGTTGTC		0.25
L07 - F	PET	TTGACGAATGAGATGAGAAGGC		0.2
L07 - R		GCTTCTTAGTGTGGCAGGATAGAAGGAG		0.2

1 µl of the extracted DNA was amplified by PCR at 13 microsatellite loci in 5 mixes (1,1 to 2,2) using a Bio-Rad thermocycler T100 (Bio-Rad, Pleasanton, CA). PCR run in a 12 µl volume containing 1.2 µl of DNA, the indicated volume for each primer and 6.4 µl of Supermix Taq-Pro COMPLETE (Denville Scientific inc., Meutchen, NJ). All PCR mixes follow the same temperature cycles: an initial step at 94°C for 60 sec, 10 cycles of 30 sec at 94°C, 45 sec at 60°C (-0.5°C per cycle) and 60 sec at 72°C, then 25 cycles of 30 sec at 94°C, 45 sec at 55°C and 60 sec at 72°C; the PCR ends by a final elongation step for 30 sec at 72 °C. The 5 PCR mixes were then combined into 2 Post-PCR mixes (1 & 2). The amplified products were separated on ABI 3500 capillary sequencer (Applied Biosystems, Foster City, CA, USA) and sized against LIZ 500 sizing standards (BioVentures, Murfreesboro, TN, USA). Controls for genotyping errors due to null alleles were analysed following the Expectation Maximization algorithm of Dempster et al. (1977) implemented in the FREE NA software (Chapuis & Estoup 2007). Additional tests of heterozygote deficiency and estimation of linkage disequilibrium were performed in GENEPOP on the Web (Rousset 2000).

Chapuis M-P, Estoup A. 2007. Mol Biol Evol. 24: 621-631.
Rousset. 2000. J Evol Biol. 13: 58-62.
Dempster AP, Laird NM, Rubin DB. 1977. J R Stat Soc B. 39:1–38. 2.
